# Use of SpyGlass™ DS II Direct Visualization System With SpyBite™ Max Forceps for the Removal of Echinococcus Granulosus Causing Biliary Obstruction

**DOI:** 10.14309/crj.0000000000001934

**Published:** 2025-12-12

**Authors:** Gandhi Lanke, Edwin Onkendi, Dauod Arif, Alli Holder, Kanak Das

**Affiliations:** 1 Department of Gastroenterology, Texas Tech University, Lubbock, TX; 2 Department of Surgery, Texas Tech University, Lubbock, TX; 3 Department of Pathology, Texas Tech University, Lubbock, TX

**Keywords:** endoscopic retrograde cholangiopancreatography, hydatid disease, SpyGlass DS II-assisted cholangioscopy, SpyBite Max forceps; echinococcus granulosus

## Abstract

Hydatid disease, also known as cystic echinococcosis, affecting the liver can vary in clinical presentation from uncomplicated to most complicated one that can rupture into the bile duct. In the past, surgery was the mainstay of treatment for biliary complications. However, with the increasing expertise of endoscopic retrograde cholangiopancreatography, biliary complications are less common. Endoscopic retrograde cholangiopancreatography can be used preoperatively and postoperatively in patients presenting with jaundice to treat biliary obstruction and biliary fistula. In our case report, we illustrate the use of cholangioscopy with SpyGlass DS II Direct Visualization System (SpyGlass DS II; Boston Scientific) SpyBite Max forceps for complete removal of the parasite from the bile duct.

## INTRODUCTION

Hydatid disease is prevalent in South America, North Africa, the Middle East, and Eastern Europe where sheep husbandry is common.^1^ Approximately 5%–25% of patients develop intracystic biliary rupture with resultant biliary obstruction and fistula.^2^ Surgery was the mainstay of treatment for biliary complications before the widespread use of endoscopic retrograde cholangiopancreatography (ERCP). By decreasing biliary tree pressure, endoscopic sphincterotomy with biliary stenting accelerates fistula closure. ERCP also allows for bile duct clearance of any intraductal Echinococcus using balloon catheter, dormia basket, and saline irrigation. SpyGlass DS II Direct Visualization System-assisted cholangioscopy aids in direct visualization of any intraductal Echinococcus and the removal of such parasite or remnant daughter vesicles from the bile duct.

## CASE REPORT

A 60-year-old Chilean man presented to the hospital for evaluation of jaundice and abdominal pain. The abdominal pain was described as epigastric, sharp, and constant with radiation to the back and without any aggravating or relieving factors. He denied any fever or chills, weight loss, and anorexia. He denied alcohol or tobacco or other recreational substances use or abuse. He grew up in a farm with various livestock in Chile. Approximately 18 years ago, he had an exploratory laparotomy in Chile for hydatid cyst removal which was complicated with the rupture of a cyst, leading to anaphylactic shock requiring endotracheal intubation and intensive care. He subsequently recovered and led normal life until recently when he developed abdominal pain and jaundice and sought further remedy.

His physical examination was only notable for icterus in both eyes and epigastric abdominal tenderness without any rigidity or guarding. Laboratory workup showed total bilirubin 9.0 mg/dL (direct bilirubin 6.6), alkaline phosphatase 297 U/L, aspartate aminotransferase 225 U/L, alanine aminotransferase 735 U/L, a negative acute viral hepatitis panel, and 2 negative sets of blood cultures.

**Figure 1.**
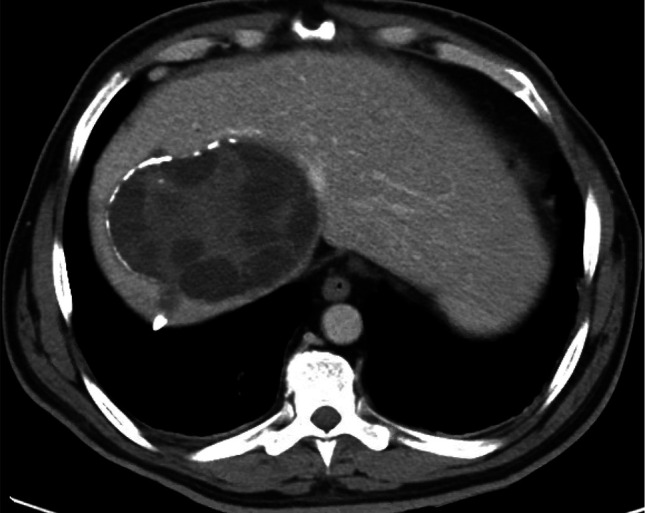
Computed tomography using a liver protocol showing hydatid cysts in the right hepatic lobe.

**Figure 2.**
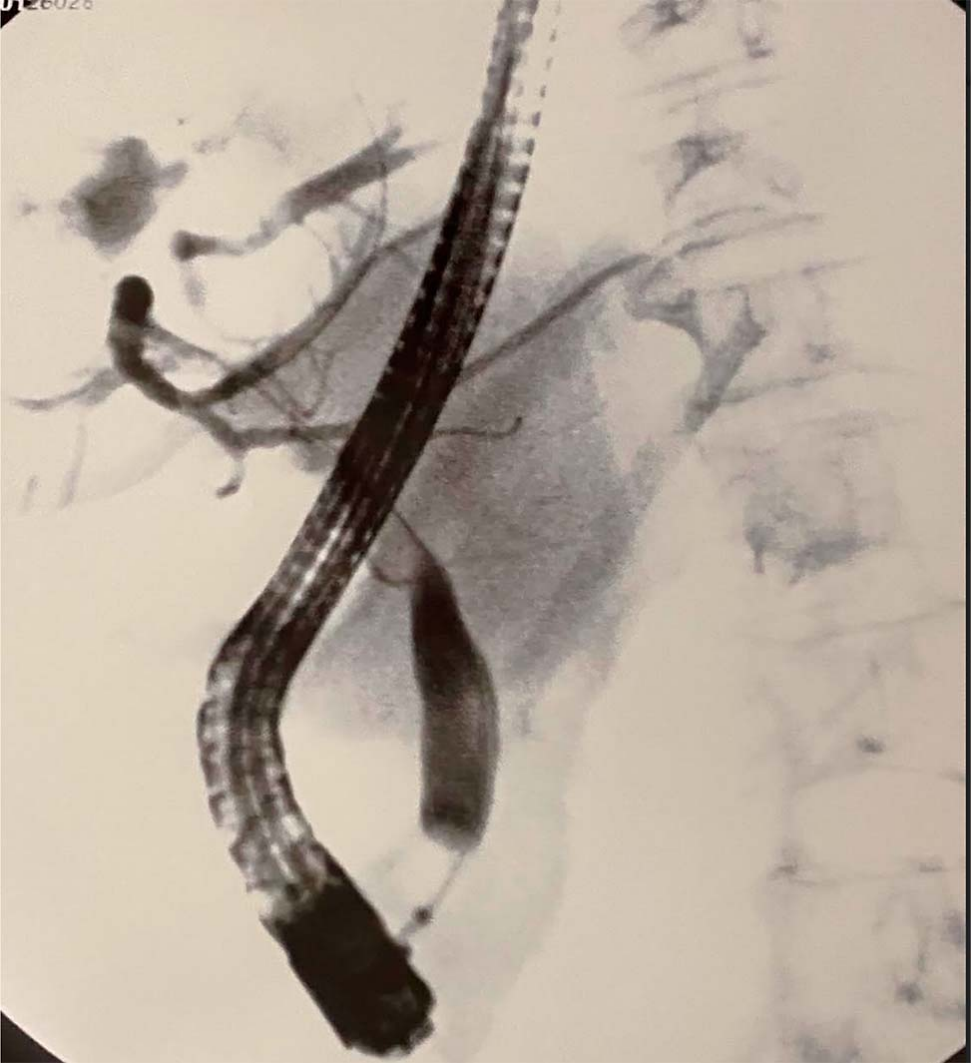
Cholangiogram showing filling defect in the common hepatic duct with dilated left-sided intrahepatic duct.

A computed tomography using a liver protocol (CT liver) showed a complex 12 cm cystic mass in the right hepatic dome with smaller (2 cm) intralesional cysts with peripheral linear areas of calcification consistent with hydatid cysts (Figure 1). A magnetic resonance cholangiopancreatography confirmed similar findings. An endoscopic ultrasonography showed an 85 mm × 89 mm multiseptated multiloculated anechoic cystic mass in the central aspect of the liver. A 4 mm echogenic object without acoustic shadowing was seen in the common bile duct (CBD) measuring 9.6 mm. ERCP showed dilated intrahepatic and extrahepatic bile ducts. A cholangiogram revealed a filling defect suspicious of Echinococcus or its remnants (Figure 2). After a biliary sphincterotomy, creamy white thick parasite or its components were retrieved with the balloon sweeps. One 10 Fr × 9 cm Advanix Duodenal bend (Boston Scientific) plastic stent was placed into the CBD to ensure continued biliary drainage. Liver enzymes improved following ERCP. Biliary cytology was unremarkable. Albendazole 400 mg orally twice daily was started.

**Figure 3.**
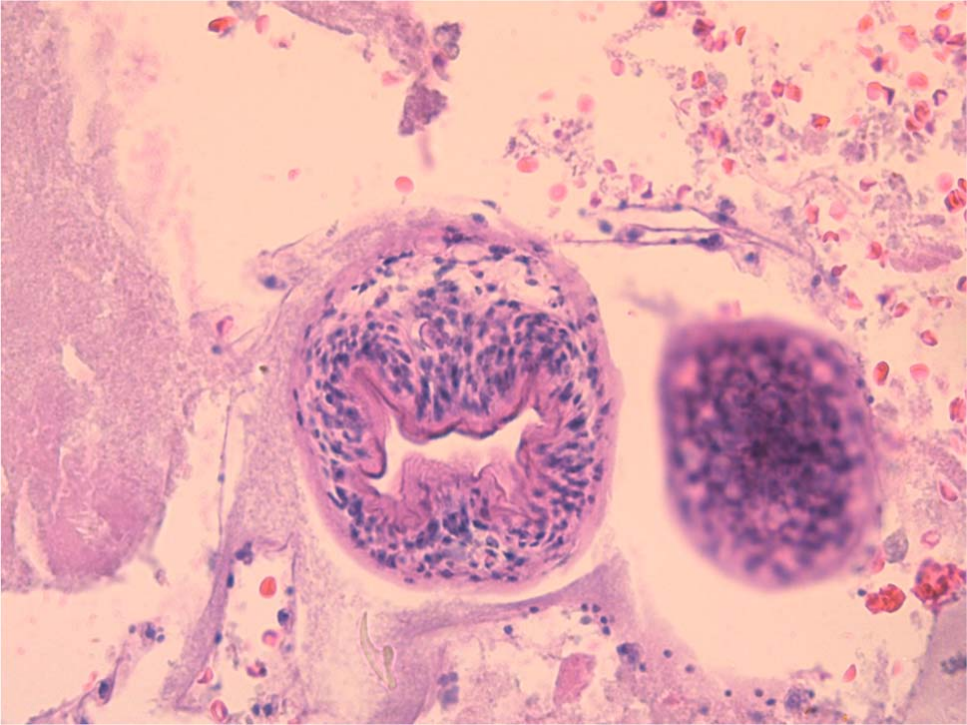
Protoscolices with sucker and refractile hooklet.

Subsequently, an open en bloc right hepatectomy with the resection of a large hydatid cyst along with the complete removal of hydatid protoscolices after an intraoperative 23% hypertonic saline sterilization of the cyst was performed. Histopathologic evaluation identified protoscolices with sucker and refractile hooklets (Figure 3).

**Figure 4.**
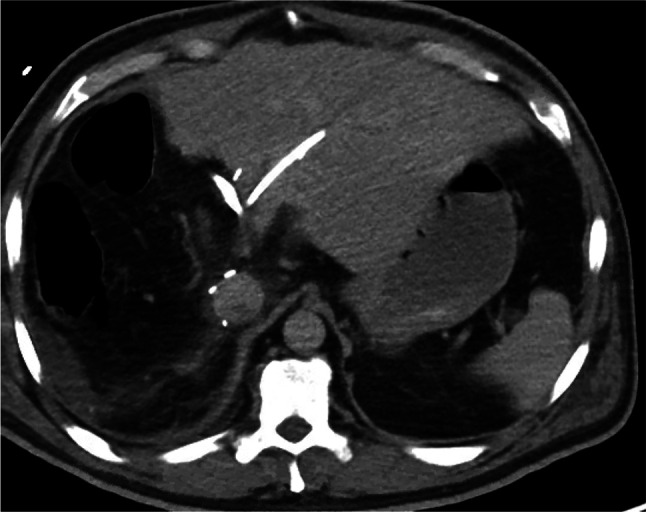
Computed tomography using a liver protocol following right hepatectomy showing appropriate postsurgical changes and biliary stent in left hepatic duct.

**Figure 5.**
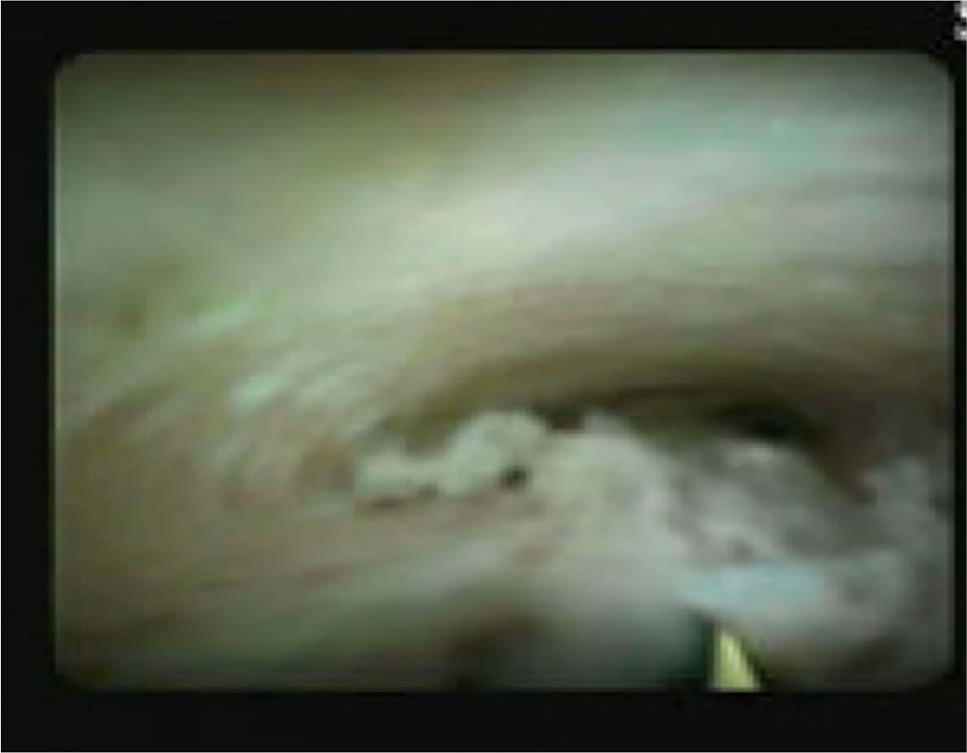
Spyglass showing Echinococcus in the bile duct.

**Figure 6.**
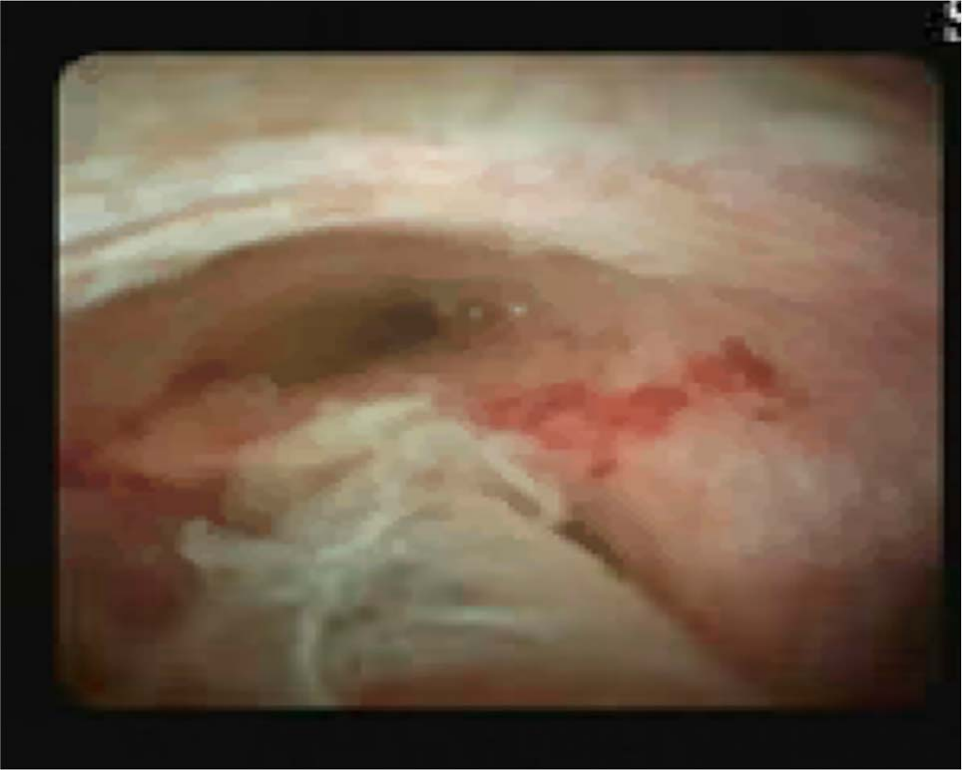
SpyBite forceps used to retrieve the Echinococcus from the bile duct.

The patient tolerated the surgery well. However, he developed a fever of 101.3 F on postoperative day 3. Piperacillin-tazobactam antibiotic was started following the collection of blood cultures that grew Enterococcus fecalis and coagulase-negative Staphylococcus. A transesophageal echocardiography as advised by infectious disease service was negative for endocarditis. Repeat CT liver protocol demonstrated interval right hepatectomy with a small 5.6 × 2.2 cm hypodense fluid collection in the operative bed (Figure 4) and appropriately positioned biliary stent without biliary ductal dilatation. At this time, an ascending cholangitis was suspected. ERCP was repeated, and CBD stent was removed. The occlusion cholangiogram showed a persistent filling defect at the common hepatic duct. SpyGlass DS II Direct Visualization System-assisted cholangioscopy revealed a creamy white thick ribbon-like structure deeply attached to the bile duct wall suspicious of an Echinococcus granulosus parasite. Appropriate saline irrigation using 0.9% sodium chloride solution (about 300–400 cc) was then performed with the application of intermittent suction forces to dislodge the parasite. This method appeared successful. Then using a SpyBite Max forceps, the structure was retrieved from the bile duct and confirmed to be Echinococcus granulosus parasite structure (Figures 5 and 6) on histopathologic assessment. The patient tolerated ERCP well. The cholangioscopy required approximately 75 minutes. No noteworthy procedural challenges were met. His follow-up blood cultures were negative. He was discharged home on a 6-week course of piperacillin-tazobactam and gentamicin. His subsequent clinic follow-up visits for 5 months were unremarkable. A follow-up CT liver protocol showed expected hypertrophy of the left lobe without any residual hydatid cyst. He was advised to follow-up in the clinic as needed.

## DISCUSSION

Hydatid disease is caused by a tape worm, Echinococcus granulosus. The 2 forms of echinococcosis are (i) cystic echinococcosis and (ii) alveolar echinococcosis.^3^ It is common in countries with more human contacts with cattle and sheep. Dogs are the definitive host. The proposed pathogenesis involves ingestion of the eggs, which eventually develop into embryos that penetrate the small intestinal mucosa to enter the portal circulation and the liver. Approximately 70% of the cysts are found in the liver.^4^

The clinical symptoms include abdominal pain, anorexia, and jaundice, usually do not appear until the cyst is at least 10 cm or occupies >70% of the liver causing compression of the bile duct.^3^

ERCP is complimentary to surgery, that once was the mainstay of treatment for biliary complications. To our knowledge, this case report is the first to illustrate the use of SpyGlass DS II-assisted cholangioscopy with SpyBite Max forceps for the extraction of biliary Echinococcus granulosus causing acute cholangitis. Reves et al used SpyGlass cholangioscopy showing bile duct wall ulceration and daughter vesicles but performed no intervention.^5^ Table 1 presents a list of previously published ERCP cases of biliary echinococcosis.

**Table 1.** Published ERCP cases of Echinococcus

Author/yr	Age (yr)	ERCP indication	Surgery	Complications
Reves et al^5^ 2024	57	Cholangitis	Hepatectomy with cyst removal	None
González-Arboleda et al^6^ 2023	36	Jaundice	Hepatic hydatid cyst removal	None
Kazzaz et al^4^ 2022	75	Obstructive jaundice	Lap cholecystectomy	None
Christodoulidis et al^7^ 2021	56	Broncho biliary fistula and, biliary rupture	Hepatic hydatid resection	Not mentioned
Guzman-Calderon et al^8^ 2020	63	Elevated liver enzymes, CBD dilation	None	None
Hamza et al^9^ 2020	28	Jaundice	Lap cholecystectomy	None
Galati et al^10^ 2006	Not mentioned	Cholangitis	Resection of the cyst wall and liver resection	Biliary fistula, cardiac arrest
Akel et al^11^ 2013	34	Cholangitis	None	None
Aday et al^12^ 2017	48	Biliary obstruction	None	None
Akcakaya et al^13^ 2006	45	Biliary fistula	None	None
Simsek et al^14^ 2003	42	Jaundice/cholangitis	Complete cyst removal	None
Giouleme et al^15^ 2001	53	Jaundice/cholangitis	Hydatid cyst removal	None

CBD, common bile duct; ERCP, endoscopic retrograde cholangiopancreatography.

Hepatic Echinococcus granulosus (Hydatid cyst) can rupture in to the bile duct and cause obstructive jaundice and biliary fistula. ERCP with SpyGlass-assisted cholangioscopy and other associated interventions can aid in the effective clearance of the daughter vesicles from the bile duct and result in complete recovery of the patient from such biliary complications.

## DISCLOSURES

Author contributions: G. Lanke composed and drafted the paper. E. Onkendi composed the paper. D. Arif pathology description and images. A. Holder specimen grossing. K. Das conceptualized, designed, revised, and edited the draft. G. Lanke is the article guarantor.

Financial disclosure: None to report.

Informed consent was obtained for this case report.

## ABBREVIATIONS:

CBD: Common bile duct
CT: Computed tomography
ERCP: Endoscopic retrograde cholangiopancreatography
